# Early Mortality Risk in Acute Trauma Patients: Predictive Value of Injury Severity Score, Trauma Index, and Different Types of Shock Indices

**DOI:** 10.3390/jcm11237219

**Published:** 2022-12-05

**Authors:** Guoyang Dai, Xin Lu, Feng Xu, Deli Xu, Pengfei Li, Xionghui Chen, Fengbao Guo

**Affiliations:** Department of Emergency Medicine, the First Affiliated Hospital of Soochow University, Suzhou 215000, China

**Keywords:** mortality risk, injury severity score, trauma index, shock index, trauma

## Abstract

Objective: This study aimed to explore the predictive value of the Injury Severity Score (ISS), Trauma Index (TI) and different types of shock indices (SI) on the early mortality risk of acute trauma patients. Methods: Clinical data of acute trauma patients who met the inclusion and exclusion criteria of this study and were treated in the hospital from January 2020 to December 2020 were retrospectively collected, including gender, age, trauma mechanism, severe injury site, ISS, TI, admission vital signs, different types of shock indices (SI), death within 7 days, length of hospital stay, and Glasgow Outcome Score (GOS). The predictive value of the Injury Severity Score, Trauma Index, and different types of shock indices on the risk of early mortality in patients with acute trauma were compared using relevant statistical methods. Results: A total of 283 acute trauma patients (mean age 54.0 ± 17.9 years, 30.74% female) were included, and 43 (15.19%) of the patients died during 7 days of hospitalization. The admission ISS, TI, SI, MSI, and ASI in the survival group were significantly lower than those in the death group, and the difference was statistically significant (*p* < 0.05). Meanwhile, different trauma assessment tools included in the study have certain predictive value for early mortality risk of trauma patients. Conclusions: The TI indicates a better capability to predict the risk of early death in patients with acute trauma. As the most sensitive predictor, the SI has the greatest reference value in predicting the risk of early death in patients with traumatic shock.

## 1. Introduction

At present, high energy traumatic events occur frequently in developing countries, which seriously affect the survival prognosis of trauma patients and the burden of family life [1]. There are about 10 million deaths due to severe trauma in the world every year, and trauma treatment around the world is facing huge challenges [2]. The concept of “golden time” has been proposed in previous studies related to trauma treatment, emphasizing that the risk of early mortality after severe trauma is increased and rapid intervention is required [3]. Therefore, the accurate assessment of the early mortality risk of acute trauma patients is of great significance to improve the efficiency and success rate of severe trauma treatment. The ideal prehospital triage tool for trauma should be simple and easy to use. By accurately distinguishing the degree of injury, severe trauma patients would be transported to high-level trauma centers while mild trauma patients to low-level trauma centers, which optimizes the use of health resources and significantly reduces the mortality of trauma patients [4]. However, in the current trauma emergency environment, there is a wide variety of prehospital triage assessment tools for trauma severity and post-traumatic blood loss. A study by Abajas Bustillo et al. showed that the Injury Severity Score (ISS), a scoring tool for severity of injury, serves as the “gold standard” for assessing injury severity, with higher scores indicating greater severity of injury and greater likelihood of post-traumatic mortality [5]. In recent years, the Trauma Index (TI) [6], the quantitative criteria for evaluating the severity of trauma, has been widely used in clinical frontline work as an objective, simple, easy-to-master, highly maneuverable, and individualized initial assessment index of trauma severity, and can predict the mortality risk and survival prognosis of acute trauma patients to a certain extent. In addition, a study by Davenport et al. identified uncontrolled post-traumatic hemorrhage as the leading cause of potentially preventable death in trauma patients [7]. Shock Index (SI) is the ratio of pulse rate to systolic blood pressure (SBP), as an important indicator for evaluating the severity of blood loss in trauma patients during pre-hospital emergency care, and it has a certain value in predicting the hospitalization probability and in-hospital mortality of trauma patients [8]. However, the evaluation of the severity of post-traumatic blood loss is often interfered with by many factors such as diastolic blood pressure (DBP), body temperature, age, and measurement position of trauma patients. Therefore, in clinical diagnosis and treatment, different types of shock index should be adopted according to the actual situation of patients to improve the accuracy of the evaluation of hemorrhagic shock in different trauma patients. The modified shock index (MSI) is the ratio of heart rate to mean blood pressure. The mean blood pressure (MAP) of emergency admission patients is calculated from systolic and diastolic blood pressure, i.e., MAP = [(DBP × 2) + SBP]/3, which can be used to evaluate bleeding severity more objectively [9]. Age-adjusted SI (ASI), by age multiplication of shock index, can to some extent accurately identify the most severely injured child or elderly person after blunt trauma, and thus predict the need for blood transfusion in trauma patients [10]. Although the above trauma triage tools have a certain value in assessing the prognosis of trauma patients, the differences in the predictive levels of early mortality risk in trauma patients are still debated. Therefore, this study retrospectively analyzed the clinical data of 283 trauma patients who met the inclusion and exclusion criteria, in order to explore the predictive value of ISS, TI, and different types of SI on the risk of early mortality in trauma patients, and to provide references for early diagnosis and treatment of trauma.

## 2. Materials and Methods

### 2.1. Clinical Information

**Figure 1:**
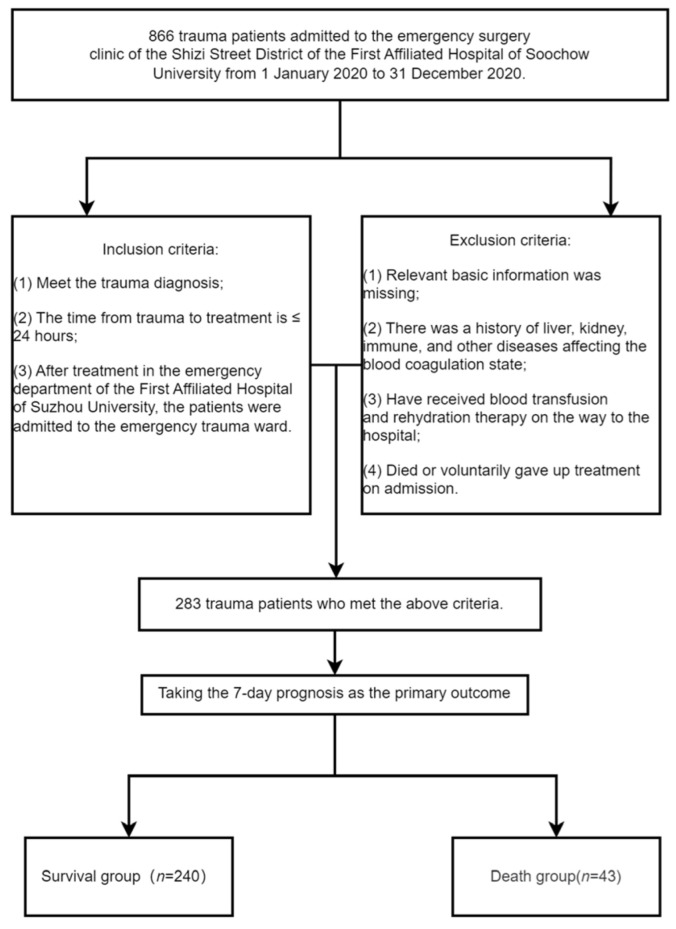
Study Grouping Criteria Flowchart.

A retrospective analysis method was used to select 866 trauma patients admitted to the emergency surgery clinic of the Shizi Street District of the First Affiliated Hospital of Soochow University from 1 January 2020 to 31 December 2020. Inclusion criteria: (1) Meet the trauma diagnosis; (2) Time from injury to hospital admission ≤ 24 h; (3) After treatment in the emergency department of the First Affiliated Hospital of Suzhou University, the patients were admitted to the emergency trauma ward. Exclusion criteria: (1) Relevant basic information was missing; (2) There was a history of liver, kidney, immune, and other diseases affecting the blood coagulation state; (3) Have received blood transfusion and rehydration therapy (that is, the trauma patient receives ≥ 500 mL infusion treatment before being admitted to the hospital to measure vital signs) on the way to the hospital; (4) Died or voluntarily gave up treatment on admission. Finally, 283 trauma patients were included, including 196 males and 87 females. The flow chart for inclusion and exclusion criteria is presented in Figure 1. All patients were admitted to the emergency trauma ward for treatment, and their survival was assessed 7 days later. The research materials have been reviewed by the Medical Ethics Committee of the First Affiliated Hospital of Soochow University, and all patients have been treated anonymously.

### 2.2. Research Design

The Emergency Department of the First Affiliated Hospital of Soochow University has now become one of the most important clinical diagnosis and treatment centers for trauma patients in Suzhou City, Jiangsu Province. We retrospectively collected the data of 283 trauma patients who met the inclusion and exclusion criteria, including basic data: gender, age, past medical history, medication history; clinical data: injury mechanism, serious injury site, ISS, TI, admission vital signs, death within 7 days, hospital stay, and Glasgow Outcome Scale (GOS) at discharge, and the SI, MSI, and SIA at admission were calculated. (1) Taking the 7-day prognosis as the primary outcome, the 283 patients included in the study were divided into a survival group (240 cases) and a death group (43 cases), and the differences in clinical data of the two groups were compared. (2) ROC curve analysis was used to compare the predictive value of different assessment tools on the risk of early mortality in acute trauma patients.

### 2.3. Statistical Methods

Statistical analysis of data was performed using SPSS 26.0 statistical software. All normally distributed measurement data are compared between two groups by independent sample *t* test, which is expressed by mean ± standard deviation, count data is expressed by chi-square test, and all cases are expressed by (*n*). The measurement data of the state distribution were expressed as the median and quartile M (QL, QU), and the Mann–Whitney U test was used. Z-statistics, defined by the area under the receiver operating curve (ROC), were compared using the nonparametric test developed by Delong et al. in MedCalc software to discriminate between trauma patients with different ISS, TI, SI, MSI, and ASI at admission to determine the value of the indicator. *p* < 0.05 was considered to be statistically significant.

## 3. Results

### 3.1. Comparison of Basic Clinical Data

A total of 283 trauma patients were included, including 196 males and 87 females, aged 15–96 years. The mean age of the study population was 54.0 ± 17.9 years. Comparing the death group (*n* = 43, 15.19%) and survival group (*n* = 240, 84.81%), there was no significant difference in gender, main injury site, and cause of injury between the two groups (*p* > 0.05), but there was statistical significance in age, systolic blood pressure, diastolic blood pressure, mean arterial pressure, pulse rate, the number of days in hospital and GOS (*p* < 0.05). The results are shown in Table 1.

### 3.2. Comparison of Different Trauma Assessment Tools

The median values of different trauma assessment tools such as ISS 13 (10, 17), TI 10 (9, 12), SI 0.66 (0.60, 0.76), MSI 0.90 (0.81, 1.05), and ASI 34.18 (25.99, 45.71) in the survival group were significantly lower than those in the death group 21 (18, 26), 20 (17, 24), 1.27 (1.13, 1.60), 1.58 (1.38, 1.82),and 81.90 (63.45, 100.62), respectively, and the difference was statistically significant (*p* < 0.05). The specific results are shown in Table 2.

### 3.3. Prediction of Early Mortality Risk in Trauma Patients by ISS, TI, SI, MSI, and ASI

**Figure 2:**
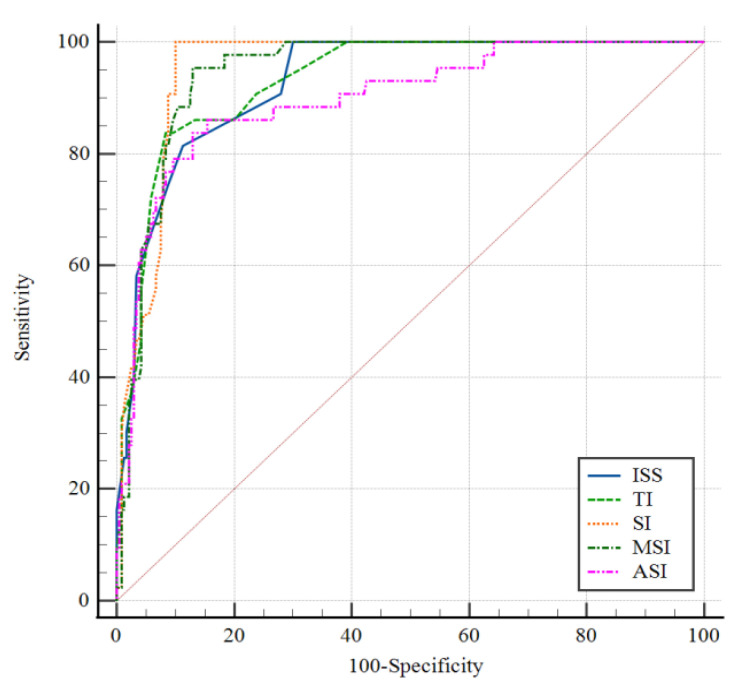
(ROC) curves of different trauma assessment tools for predicting the risk of early mortality in patients.

The ROC curve was used to analyze the prediction of ISS, TI, SI, MSI, and ASI on the risk of early mortality in trauma patients. The area under the ROC of ISS, TI, SI, MSI, and ASI were 0.929 (95% CI 0.892–0.956), 0.932 (95% CI 0.896–0.958), 0.953 (95%CI 0.921–0.975), 0.945 (95% CI 0.911–0.968), and 0.899 (95% CI 0.858–0.931), respectively, and the detailed results are shown in Figure 2. The above different trauma assessment tools have a certain predictive value for the early mortality risk of trauma patients. After statistical analysis, the Z-statistic value of the SI (Z = 39.025) is significantly higher than that of the ISS (Z = 25.674), TI (Z = 25.199), MSI (Z = 33.412), and ASI (Z = 14.690). The detailed results are shown in Table 3. Therefore, the SI has a better ability to predict the severity of early injury in patients with trauma than ISS. SI, as the most sensitive predictor, has the greatest reference value in the evaluation of traumatic shock patients.

## 4. Discussion

Acute trauma is characterized by complex disease, a high disability rate and fatality rate, and it is difficult to treat. Currently, it is the main cause of death for people under the age of 45 [11]. Therefore, in order to improve the efficiency and quality of trauma diagnosis and treatment and reduce the mortality risk of acute trauma patients, it is particularly important to quickly and accurately assess the severity of acute trauma patients, triage trauma patients in a timely manner, and then take effective treatment measures. The main finding of our study is that the ISS, TI, SI, MSI, and ASI can all predict the risk of early mortality in acute trauma patients to a certain extent, and the higher the value, the greater the risk of mortality. Among them, the predictive value of SI for acute trauma mortality risk is better than other research indicators.

The current general ISS still uses the latest version of the Abbreviated Injury Scale (AIS) in 2005 as the anatomical basis to assess the severity of injury in trauma patients [12]. Early studies have shown that the fatality rate of trauma patients with an ISS ≥ 16 points is about 10%, and an ISS ≥ 16 is widely used as a criterion for judging multiple traumas [13]. In addition, previous studies have reported that the ISS cut-off value in severe trauma patients may be >16, >17, >18, or other values [14,15]. However, the current cut-off value for the evaluation of multiple traumas using the ISS may be controversial. We analyzed the ISS for early mortality risk prediction in 283 trauma patients, and found that the best correlation criterion for the ISS was >17, which was consistent with the results of previous studies. However, the shortcomings of this study are that the sample size is small, and the analysis results lack certain authority. So, in order to further explore the prediction ability of ISS, a large-scale statistical analysis of data is required in the future. The TI evaluates acute trauma including injury type, injury site, consciousness, respiration, and circulation. Compared with ISS, the lower the TI, the safer the patient, which is consistent with the results obtained in this study. It can be seen from the above results that the TI has more advantages than the ISS in terms of sensitivity, specificity, and related criteria in assessing the risk of early mortality in acute trauma patients. The TI, as an objective, simple, easy to grasp, highly maneuverable and individualized initial assessment index of trauma severity, has been widely used in pre-hospital trauma emergency care [6]. This study concluded that a TI > 16 can be used as one of the important indicators to predict the early mortality risk of acute trauma patients. In the extremely busy and stressful emergency environment, the simple and easy-to-master TI is more convenient and reasonable to use than the ISS evaluation index. However, due to the small sample size of this study, the evaluation value of relevant indicators still needs to be continuously verified after the trauma database is perfected.

The shock index is an important index to evaluate the hemodynamic stability of the body. By dividing the pulse rate by the systolic blood pressure, SI can predict the probability of major post-traumatic hemorrhage, which is convenient for emergency doctors to take timely and effective treatment measures and improve the success rate of severe trauma treatment [16,17]. A study by Jouini et al. found that SI ≥ 1 was a predictor of 7-day and 1-month in-hospital mortality in severely traumatized patients [18]. In this study, through the statistical analysis of the 7-day death of acute trauma patients, the SI of the death group and the survival group were compared, and it was found that the median SI value of the death group was 1.27, which was significantly higher than that of the survival group, 0.66. The predictive value of predicting the risk of early mortality in acute trauma patients was >0.95, with the highest sensitivity and the highest AUC value. It can be seen that SI is of great reference value in predicting the risk of early mortality and hospitalization in patients with acute trauma [19]. When trauma patients are in a state of hypovolemia, compensatory mechanisms are activated to constrict the arteries to maintain a relatively “normal” systolic blood pressure, which makes the SI biased in the assessment of injury severity and prognosis in trauma patients. Diastolic blood pressure is less affected by the body’s compensatory mechanism, so this study included diastolic blood pressure in the calculation of mean arterial pressure, and the MSI was obtained by dividing the pulse rate by the mean arterial pressure. Using the MSI to predict the risk of early traumatic death, we found that the MSI has high predictive sensitivity and specificity, and it has important predictive value for patients with post-traumatic hypovolemia. This is consistent with previous studies [9]. In addition, when assessing the severity of trauma patients, since blood pressure and heart rate are affected by age, the accuracy of the SI in determining whether a patient is in shock and the cut-off value of the degree of shock varies among different ages. Therefore, the age shock index was introduced in this study and the effect of age on blood pressure and heart rate was excluded. The ASI was associated with higher mortality when trauma patients were >50 years old. The study by Kim et al. included 45,880 elderly trauma patients > 65 years old and found that the ASI of the emergency death group was significantly greater than that of the survival group [20]. Additionally, in this study, the average age of the patients in the death group and the survival group was 60.3 years and 52.9 years old, respectively. Compared with the survival group, the ASI was significantly higher in the death group, and there was a statistical difference between the two. However, this study is a small sample study, and the comparison of the predictive values of the SI, MSI, and ASI are still controversial, and a large amount of clinical data still needs to be further analyzed.

In summary, the ISS, TI, SI, MSI, and ASI are important predictors of the severity of acute trauma patients and the risk of early mortality. Reasonable selection and use of these scoring tools can help emergency responders to accurately and efficiently evaluate and transfer patients, and provide evidence for clinical decision-making in the treatment of trauma patients, which might contribute to reduce the mortality rate.

## 5. Conclusions

In this study, we compared the ability of the ISS, TI, SI, MSI, and ASI to predict the risk of early mortality in patients with acute trauma. Our study shows that the TI indicates a better capability to predict the risk of early death in patients with acute trauma. As the most sensitive predictor, the SI has the greatest reference value in predicting the risk of early death in patients with traumatic shock. At the same time, the SI, as an evaluation index to measure the blood loss after trauma, can derive new indicators such as the MSI and ASI under different trauma conditions, and all three have a good predictive ability on the early mortality risk of patients with acute trauma.

## Figures and Tables

**Table 1:** Comparison of basic clinical data between survivor group and death group.

Variable	Survival Group (*n* = 240)	Death Group (*n* = 43)	*p* Value
Gender			0.937 ^b^
male (*n*, %)	166 (69.17%)	30 (69.77%)	
female (*n*, %)	74 (30.83%)	13 (30.23%)	
Age (years)	52.9 ± 17.8	60.3 ± 17.5	0.013 ^a^
Main injury site			
head and neck (*n*, %)	147 (61.25%)	32 (74.42%)	0.099 ^b^
face (*n*, %)	10 (4.17%)	1 (2.33%)	0.540 ^b^
chest (*n*, %)	24 (10.00%)	4 (9.30%)	0.887 ^b^
abdomen (*n*, %)	7 (2.92%)	2 (4.65%)	0.571 ^b^
limbs and pelvis (*n*, %)	47 (19.58%)	4 (9.30%)	0.106 ^b^
body surface (*n*, %)	5 (2.08%)	0 (0.00%)	0.436 ^b^
Cause of injury			
motor vehicle accident (*n*, %)	163 (67.92%)	34 (79.07%)	0.143 ^b^
fall from height (*n*, %)	50 (20.83%)	4 (9.30%)	0.076 ^b^
crushed by heavy objects (*n*, %)	8 (3.33%)	1 (2.33%)	0.718 ^b^
other reasons (*n*, %)	19 (7.92%)	4 (9.30%)	0.763 ^b^
Systolic blood pressure (mmHg)	127 (118, 137)	89 (76,98)	<0.001 ^c^
Diastolic blood pressure (mmHg)	77 (70, 80)	65 (58,75)	<0.001 ^c^
Mean arterial pressure (mmHg)	93 (87, 100)	74 (68,80)	<0.001 ^c^
Pulse rate (times/min)	84 (78, 95)	117 (105, 134)	<0.001 ^c^
The number of days in hospital	15 (10,20)	3 (2,5)	<0.001 ^c^
Glasgow outcome scale	5 (4, 5)	1 (1,1)	<0.001 ^c^

Note: ^a^ is Mean ± sd, using independent sample *t* test; ^b^ is enumeration data, using chi-square test, ^c^ is M (QL, QU), using Mann–Whitney U test.

**Table 2:** Comparison of different trauma assessment tools between survival and death groups.

Types	Survival Group (*n* = 240)	Death Group (*n* = 43)	*p* Value
ISS	13 (10, 17)	21 (18, 26)	<0.001
TI	10 (9, 12)	20 (17, 24)	<0.001
SI	0.66 (0.60, 0.76)	1.27 (1.13, 1.60)	<0.001
MSI	0.90 (0.81, 1.05)	1.58 (1.38, 1.82)	<0.001
ASI	34.18 (25.99, 45.71)	81.90 (63.45, 100.62)	<0.001

Note: ISS: injury severity score, TI: trauma index, SI: shock index, MSI: modified shock index, ASI: age shock index.

**Table 3:** The predictive value of different trauma assessment tools on the risk of early mortality in trauma patients.

Predictors	Associated Criterion	Sensitivity (%)	Specificity (%)	AUC	SE	95%CI	YI
ISS	>17.0	81.40	88.75	0.929	0.0167	0.892–0.956	0.7015
TI	>16.0	83.72	91.67	0.932	0.0171	0.896–0.958	0.7539
SI	>0.95	100.00	90.00	0.953	0.0116	0.921–0.975	0.9000
MSI	>1.18	95.35	87.08	0.945	0.0133	0.911–0.968	0.8243
ASI	>52.7	83.72	87.08	0.899	0.0272	0.858–0.931	0.7080

Note: ISS: injury severity score, TI: trauma index, SI: shock index, MSI: modified shock index, ASI: age shock index, AUC: area under the curve, SE: standard error CI: confidence interval. YI: Youden index.

## Data Availability

The datasets used and/or analyzed during the current study are available from the corresponding author upon reasonable request.

## Abbreviations

ISS: injury severity score, TI: trauma index, SI: shock index, MSI: modified shock index, ASI: age shock index, AUC: area under the curve, SE: standard error CI: confidence interval. YI: Youden index.

## References

[1] Shanthakumar D., Payne A., Leitch T., Alfa-Wali M. (2021). Trauma Care in Low-and Middle-Income Countries. Surg. J..

[2] Naghavi M., Abajobir A.A., Abbafati C., Abbas K.M., Abd-Allah F., Abera S.F., Aboyans V., Adetokunboh O., Afshin A., Agrawal A. (2017). Global, regional, and national age-sex specific mortality for 264 causes of death, 1980–2016: A systematic analysis for the Global Burden of Disease Study 2016. Lancet.

[3] Gauss T., Ageron F.X., Devaud M.L., Debaty G., Travers S., Garrigue D., Raux M., Harrois A., Bouzat P., French Trauma Research Initiative (2019). Association of Prehospital Time to In-Hospital Trauma Mortality in a Physician-Staffed Emergency Medicine System. JAMA Surg..

[4] Voskens F.J., van Rein E.A.J., van der Sluijs R., Houwert R.M., Lichtveld R.A., Verleisdonk E.J., Segers M., van Olden G., Dijkgraaf M., Leenen L.P.H. (2018). Accuracy of Prehospital Triage in Selecting Severely Injured Trauma Patients. JAMA Surg..

[5] Abajas Bustillo R., Amo Setien F.J., Ortego Mate M.D.C., Segui Gomez M., Dura Ros M.J., Leal Costa C. (2020). Predictive capability of the injury severity score versus the new injury severity score in the categorization of the severity of trauma patients: A cross-sectional observational study. Eur. J. Trauma Emerg. Surg..

[6] Ruan H., Ge W., Li B., Zhu Y., Yang F. (2015). The application of a trauma index to assess injury severity and prognosis in hospitalized patients with acute trauma. Int. J. Clin. Exp. Med..

[7] Davenport R.A., Brohi K. (2016). Cause of trauma-induced coagulopathy. Curr. Opin. Anaesthesiol..

[8] Balhara K.S., Hsieh Y.H., Hamade B., Circh R., Kelen G.D., Bayram J.D. (2017). Clinical metrics in emergency medicine: The shock index and the probability of hospital admission and inpatient mortality. Emerg. Med. J..

[9] Liu Y.-C., Liu J.-H., Fang Z.A., Shan G.-L., Xu J., Qi Z.-W., Zhu H.-D., Yu X.-Z. (2012). Modified shock index and mortality rate of emergency patients. World J. Emerg. Med..

[10] Rau C.S., Wu S.C., Kuo S.C., Spencer C.H., Pao-Jen K., Shiun-Yuan H., Chen Y.-C., Hsieh H.-Y., Hsieh C.-H., Liu H.-T. (2016). Prediction of Massive Transfusion in Trauma Patients with Shock Index, Modified Shock Index, and Age Shock Index. Int. J. Environ. Res. Public Health.

[11] Waits C.M.K., Bower A., Simms K.N., Feldman B.C., Kim N., Sergeant S., Chilton F.H., VandeVord P.J., Langefeld C.D., Rahbar E. (2020). A Pilot Study Assessing the Impact of rs174537 on Circulating Polyunsaturated Fatty Acids and the Inflammatory Response in Patients with Traumatic Brain Injury. J. Neurotrauma.

[12] Schellenberg M., Owattanapanich N., Grigorian A., Lam L., Nahmias J., Inaba K. (2021). Surviving Nonsurvivable Injuries: Patients Who Elude the ‘Lethal’ Abbreviated Injury Scale Score of Six. J. Surg. Res..

[13] Butcher N.E., Balogh Z.J. (2014). Update on the definition of polytrauma. Eur. J. Trauma Emerg. Surg..

[14] Bone L.B., McNamara K., Shine B., Border J. (1994). Mortality in multiple trauma patients with fractures. J. Trauma.

[15] Hildebrand F., Giannoudis P., Kretteck C., Pape H.-C. (2004). Damage control: Extremities. Injury.

[16] El-Menyar A., Goyal P., Tilley E., Latifi R. (2018). The clinical utility of shock index to predict the need for blood transfusion and outcomes in trauma. J. Surg. Res..

[17] Olaussen A., Blackburn T., Mitra B., Fitzgerald M. (2014). Review article: Shock index for prediction of critical bleeding post-trauma: A systematic review. Emerg. Med. Australas..

[18] Jouini S., Jebali A., Hedhli H., Ben Kaddour R., Mrabet A., Hebaieb F. (2019). Predictive value of shock index ≥1 in severe trauma patients in emergency department. Tunis. Med..

[19] Vang M., Østberg M., Steinmetz J., Rasmussen L.S. (2022). Shock index as a predictor for mortality in trauma patients: A systematic review and meta-analysis. Eur. J. Trauma Emerg. Surg..

[20] Kim S.Y., Hong K.J., Shin S.D., Ro Y.S., Ahn K.O., Kim Y.J., Lee E.J. (2016). Validation of the Shock Index, Modified Shock Index, and Age Shock Index for Predicting Mortality of Geriatric Trauma Patients in Emergency Departments. J. Korean Med. Sci..

